# Antiferromagnetic Ordering and Transport Anomalies in Single-Crystalline CeAgAs_2_

**DOI:** 10.3390/ma13173865

**Published:** 2020-09-01

**Authors:** Maria Szlawska, Daniel Gnida, Piotr Ruszała, Maciej J. Winiarski, Małgorzata Samsel-Czekała, Marcus Schmidt, Yuri Grin, Dariusz Kaczorowski

**Affiliations:** 1 Institute of Low Temperature and Structure Research, Polish Academy of Sciences, 50-950 Wrocław, Poland; d.gnida@intibs.pl (D.G.); p.ruszala@intibs.pl (P.R.); m.winiarski@intibs.pl (M.J.W.); m.samsel@intibs.pl (M.S.-C.); d.kaczorowski@intibs.pl (D.K.); 2 Max-Planck-Institut für Chemische Physik Fester Stoffe, 01187 Dresden, Germany; marcus.schmidt@cpfs.mpg.de (M.S.); grin@cpfs.mpg.de (Y.G.)

**Keywords:** cerium pnictides, antiferromagnetism, metamagnetism, Kondo effect, pseudo-gap

## Abstract

Single crystals of the ternary cerium arsenide CeAgAs_2_ were grown by chemical vapor transport. They were studied by means of x-ray diffraction, magnetization, heat capacity and electrical transport measurements. The experimental research was supplemented with electronic band structure calculations. The compound was confirmed to order antiferromagnetically at the Néel temperature of 4.9 K and to undergo metamagnetic transition in a field of 0.5 T at 1.72 K. The electrical resistivity shows distinct increase at low temperatures, which origin is discussed in terms of pseudo-gap formation in the density of states at the Fermi level and quantum corrections to the resistivity in the presence of atom disorder due to crystal structure imperfections.

## 1. Introduction

Most of compounds with composition CeTX_2_, where T is a transition metal and X stands for a pnictogen, crystallize with the tetragonal HfCuSi_2_-type structure (space group P4/nmm) or its derivatives. They were reported to show large variety of physical behaviors, e.g., field-induced quantum criticality in CeAuSb_2_ [1], strongly anomalous low-temperature characteristics in CeCuAs_2_ [2], or different types of magnetic ordering [3,4,5,6].

The formation of CeAgAs_2_ was first established by Demchyna et al. [7] and later confirmed by Eschen and Jeitschko [8]. Initially, the compound was reported to adopt the tetragonal HfCuSi_2_-type unit cell [8], yet more advanced investigations of single crystals of CeAgAs_2_ revealed that actually it has an orthorhombic unit cell, which can be described as a slightly distorted derivative of the HfCuSi_2_ structure (s.g. *Pmca*) [9]. A characteristic feature of this structure is the presence of cis-trans chains of As atoms, which brings about breaking of the tetragonal symmetry and thus orthorhombic deformation. As the symmetry reduction occurs just because of a small shift of one half of the As atoms [9,10,11], the tetragonal symmetry is widely preserved and the compound shows hardly any anisotropy in properties within the *ab* plane [11,12].

In the first report on the magnetic properties of CeAgAs_2_, the compound was characterized as an antiferromagnet (AFM) with the Néel temperature $T_{N}$ of 5.5 K, and a metamagnetic (MM) transition in a field of 0.46 T at the temperature T=1.8 K [9]. Subsequent investigations, performed on powder samples, showed that CeAgAs_2_ undergoes two AFM-like transitions at $T_{N1}=6$ K and $T_{N2}=4.8$ K [6]. The neutron diffraction experiments confirmed the AFM ordering below $T_{N}=6\;K$ and the MM transition in a field of $\mu_{0}H=0.3\;T$ in the temperatures between 4 and 6 K [11]. The Ce magnetic moments, being confined to the *ab* plane of the orthorhombic unit cell, were found to be coupled ferromagnetically in that plane, and antiferromagnetically along the *c* axis [11]. The staggered magnetic moment was established to be 1 $\mu_{B}$ [11]. The most recent paper by Mondal et al. on the bulk physical properties of single-crystalline CeAgAs_2_ re-established the existence of double magnetic transition at low temperatures, namely at 6 and 4.9 K, and highlighted the role of both crystalline electric field (CEF) interactions and Kondo effect in governing the low-temperature magnetic and transport characteristics of the compound [12].

Here, we report on investigations of high quality single crystals of CeAgAs_2_, done in parallel and fully independent of Mondal et al. Remarkably, our results somewhat differ from those reported by the latter authors, and hence may challenge the hitherto understanding of the actual nature of the electronic ground state in CeAgAs_2_.

## 2. Results

### 2.1. Crystal Structure

The details on the refinement and the crystallographic information are given in Table 1, Table 2 and Table 3. Most importantly, the orthorhombic distortion reported in Ref. [9] was confirmed by the difference in the lattice parameters and by different extinction conditions in the 0kl and hk0 zones. Furthermore, in line with the findings communicated in Refs. [9,10], the intensities of the single crystal diffraction data of the investigated crystal were calculated as a sum of two diffraction domains related by the twin matrix (001;0–10;100), i.e., the [010] direction of the diffraction domains is preserved, while the [100] and [001] directions are interchanged due to pseudo-tetragonal unit cell. For that reason, the physical properties measurements were performed parallel and perpendicular to [010] direction.

The EDX measurements indicated that the prepared crystal was homogeneous and single phase. The EDX analysis yielded the composition Ce – 24.6(4) at.%, Ag – 25.0(3) at.%, As – 50.4(2) at.%, that corresponds well to the nominal one.

### 2.2. Magnetic Properties

**Figure 1:**
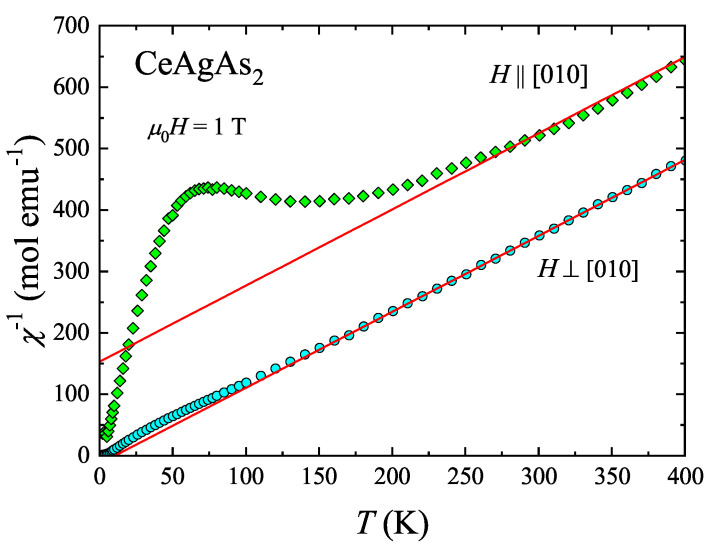
Temperature dependencies of the reciprocal magnetic susceptibility of single-crystalline CeAgAs_2_, measured at constant field oriented parallel and perpendicular to [010] direction. The solid lines represent the fits of the Curie–Weiss law to the experimental data, respectively.

Figure 1 presents the temperature dependencies of the reciprocal molar magnetic susceptibility of CeAgAs_2_, $\chi_{\|}$ and $\chi_{⊥}$, measured parallel and perpendicular to [010] direction, respectively. The compound exhibits large magnetocrystalline anisotropy and the $\chi_{\|}^{-1}\left(T\right)$ variation shows a wide maximum at about 70 K. At high temperatures (above 50 K and 250 K for susceptibility variation taken with H⊥c and H‖c, respectively), both curves follow Curie–Weiss (C-W) law in the form $\chi =\frac{C}{T-\theta_{P}}$. The least-square fitting parameters of this formula to the experimental data obtained with H⊥c are $\mu_{\mathrm{eff}}^{⊥}=\sqrt{8C}=2.54\;\mu_{B}$ and $\theta_{P}^{⊥}=10\;K$. The magnetic effective moment $\mu_{\mathrm{eff}}$ is the same as that predicted within Russel–Saunders coupling scenario for a free trivalent Ce ion (2.54 $\mu_{B}$). In turn, the positive sign of the paramagnetic Curie temperature $\theta_{P}$ hints at importance of ferromagnetic interactions, as inferred from neutron diffraction data [11]. Due to rather narrow range of straight-linear variation of $\chi_{\|}\left(T\right)$, the effective magnetic moment was assumed to be the same as for H⊥c, while fitting the C-W dependence. The obtained value of $\theta_{P}^{\|}$ is equal to −123 K. The large difference between $\theta_{P}$ estimated for the two main crystallographic directions is a measure of strong magnetocrystalline anisotropy. The large absolute value of $\theta_{P}^{\|}$ likely results from CEF splitting of the J=5/2 multiplet into three Kramer’s doublets.

**Figure 2:**
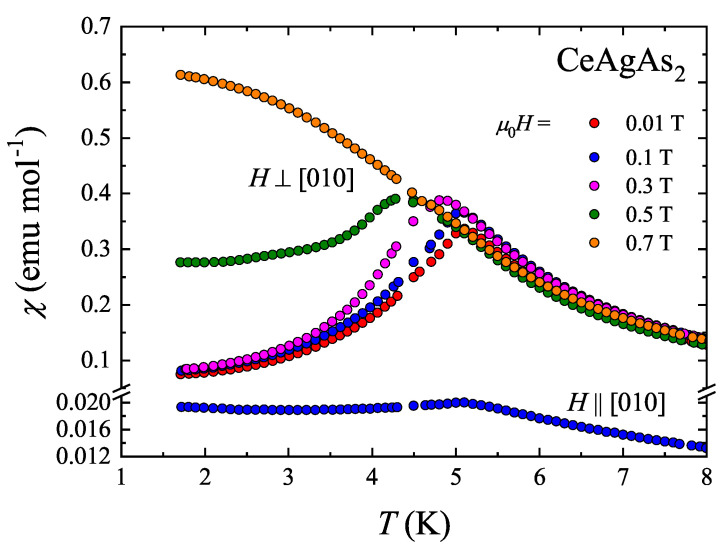
Temperature dependencies of the molar magnetic susceptibility of single crystalline CeAgAs_2_ measured in a magnetic field of different magnitude oriented perpendicular to the crystallographic [010] axis and in a field of 0.1 T applied parallel to the [010] axis.

The low-temperature variations of the magnetic susceptibility of CeAgAs_2_ measured in a few different magnetic fields oriented within the (010) plane and in a field of 0.1 T applied along [010] are presented in Figure 2. The susceptibility shows large magnetic anisotropy with the component taken along [010] being much smaller than that taken within the (010) plane. This result is consistent with the neutron diffraction experiment, that revealed that the magnetic moments in CeAgAs_2_ are confined to the (010) plane [11]. The $\chi_{⊥}\left(T\right)$ variations measured in weak fields exhibit distinct maxima, which signal the onset of long-range AFM ordering. The transition temperature defined as an inflection point on the $\chi_{⊥}T\left(T\right)$ variation taken at 0.01 T (Ref. [13]) amounts to 4.9 K. This value is somewhat smaller than those reported in Refs. [9,11] but the same as that of the lower-temperature transition in Refs. [6,12]. In contrast to the behavior of $\chi_{⊥}\left(T\right)$, no maximum is seen in the $\chi_{\|}\left(T\right)$ variation (some little effect can be attributed to small misalignment of the specimen), and in this case the susceptibility shows below $T_{N}$ a plateau characteristic for antiferromagnets probed perpendicular to the direction of magnetic moments.

With increasing the magnetic field strength, the maximum in $\chi_{⊥}\left(T\right)$ shifts towards lower temperatures, as may be expected for antiferromagnets. In fields stronger than 0.5 T, the maximum is no longer discernible suggesting the occurrence of the field-induced MM transition.

**Figure 3:**
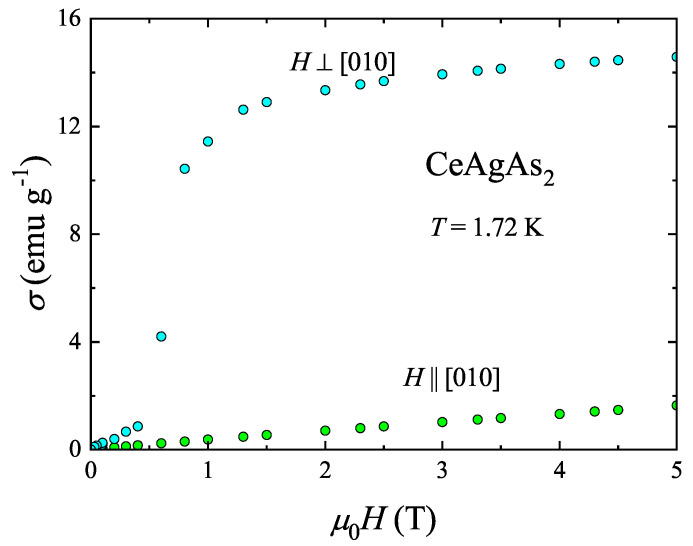
Field dependencies of the magnetization of single-crystalline CeAgAs_2_ measured at 1.72 K in magnetic field oriented along and perpendicular to [010].

The MM character of CeAgAs_2_ is corroborated by inspecting the field dependencies of the magnetization measured in the ordered state. The isotherms taken at 1.72 K are presented in Figure 3. The variation measured along [010], being clearly the hard magnetic direction, is linear up to a limiting field of 5 T. In contrast, the magnetization measured with the magnetic field applied perpendicular to [010] shows a rapid increase near 0.5 T, and then tends towards saturation in stronger fields. The magnetic moment measured at 5 T has a value of 1 $\mu_{B}$, in perfect agreement with the neutron diffraction data [11].

### 2.3. Heat Capacity

**Figure 4:**
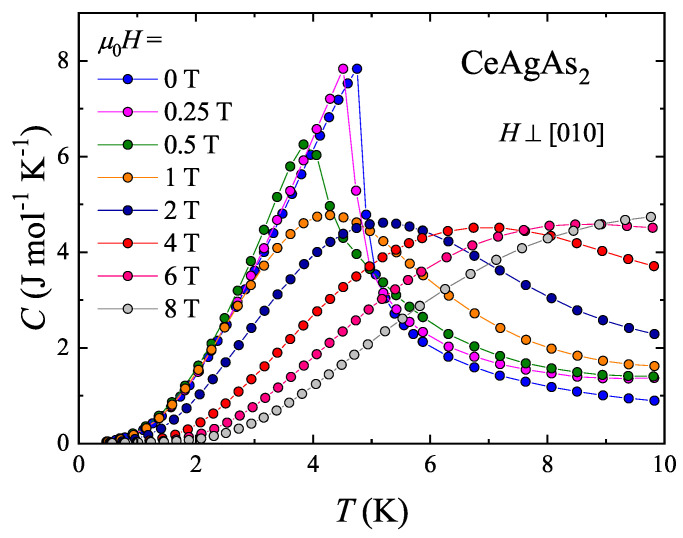
Low temperature specific heat data of single-crystalline CeAgAs_2_ measured in zero field and in several finite magnetic fields applied within the (010) plane of the orthorhombic unit cell.

The low-temperature dependencies of the specific heat of CeAgAs_2_ measured in various external magnetic fields applied within the (010) plane are shown in Figure 4. In zero field C(T) exhibits a pronounced λ-like anomaly at the Néel temperature $T_{N}=4.9$ K, in concert with the magnetic data. Upon gradual increasing the magnetic field strength, this anomaly initially shifts towards lower temperatures, as expected for antiferromagnets, and in fields stronger than 0.5 T, the maximum in C(T) broadens and moves towards higher temperatures, in a ferromagnetic-like manner. The observed behavior corroborates the MM-like phase transition anticipated from the magnetization data.

**Figure 5:**
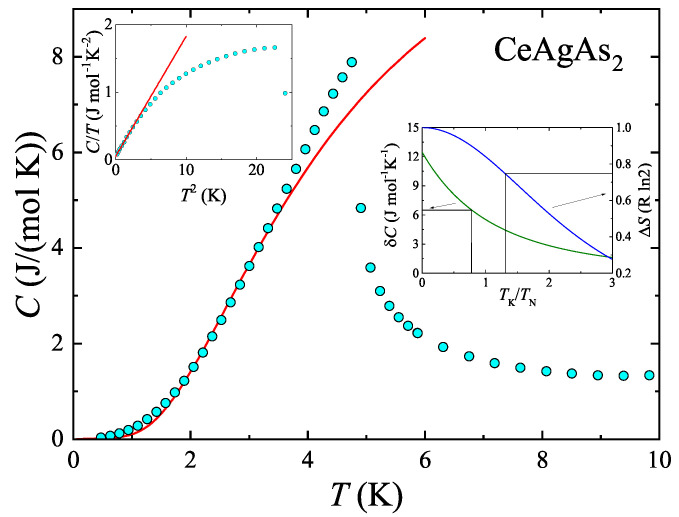
Zero-field low-temperature dependence of the magnetic contribution to the specific heat of CeAgAs_2_. Solid line represents the function described in the text. The left-hand side inset presents the $C/TvsT^{2}$ dependence. The right-hand side inset visualizes the methods used to estimate the Kondo temperature (see the text).

Figure 5 presents the zero-field dependence of the specific heat of CeAgAs_2_. As can be inferred from the figure, below $T_{N}$, the C(T) variation can be well described by the equation [14,15]

(1) $$C_{\mathrm{mag}}=\gamma_{4f}T+aT^{-0.5}\exp\left(\frac{-\Delta }{T}\right),$$

where the first term accounts for the electronic contribution and the second one is appropriate for excitations of AFM spin waves above a gap Δ in the magnon spectrum (phonon contribution was considered to be negligible at such low temperatures). The electronic part of the specific heat was estimated by linear fit of the C/T vs $T^{2}$ variation at lowest temperatures (see the inset to Figure 5) as $\gamma_{4f}=50$ mJ mol${}^{-1}$ K ${}^{-2}$. The least-square fit of Equation (1) to the experimental data with fixed $\gamma_{4f}$ value yielded the parameters: $a=64.7\;J\;{\mathrm{mol}}^{-1}K^{-0.5}$ and Δ=7.1K. The moderately enhanced value of $\gamma_{4f}$, being close to that given in Ref. [12], manifests the importance of strong electronic correlations in the compound studied. Remarkably, the energy gap Δ has magnitude similar to the Néel temperature of CeAgAs_2_.

In AFM Kondo lattices, specific heat jump due to magnetic ordering δC is reduced compared to the value predicted within the mean-field approximation. In the framework of the S=1/2 resonant model, δC is related to the characteristic Kondo temperature $T_{K}$ via the formula [16,17,18]

(2) $$\delta C=\frac{6k_{B}}{\psi^{\prime \prime \prime }\left(\frac{1}{2}+\zeta \right)}{\left[\psi^{\prime }\left(\frac{1}{2}+\zeta \right)+\zeta \psi^{\prime \prime }\left(\frac{1}{2}+\zeta \right)\right]}^{2},$$

where $\zeta =(T_{K}/T_{N})/2\pi$, while $\psi^{\prime }$, $\psi^{\prime \prime }$ and $\psi^{\prime \prime \prime }$ are first three derivatives of the digamma function. The inset to Figure 5 shows this universal relation that implied $T_{K}$ of about 3.8 K for the jump 6.5 $J\;{\mathrm{mol}}_{\mathrm{Ce}}^{-1}K^{-1}$, observed for CeAgAs_2_.

Another way of determining $T_{K}$ in Kondo lattices is based on the analysis of magnetic entropy released by the Néel temperature $T_{N}$, which can be expressed as [19]

(3) $$\Delta S\left(T_{N}\right)=R\left(\ln\left[1+\exp\left(\frac{-T_{K}}{T_{N}}\right)\right]\frac{T_{K}}{T_{N}}\frac{\exp(\frac{-T_{K}}{T_{N}})}{1+\exp(\frac{-T_{K}}{T_{N}})}\right).$$

For CeAgAs_2_ one finds $\Delta S(T_{N})=0.75\;R\ln2$, which would imply the Kondo temperature of about 6.4 K (see the inset to Figure 5). The so-derived values of $T_{K}$ are larger than that given in Ref. [12].

### 2.4. Magnetotransport Properties

**Figure 6:**
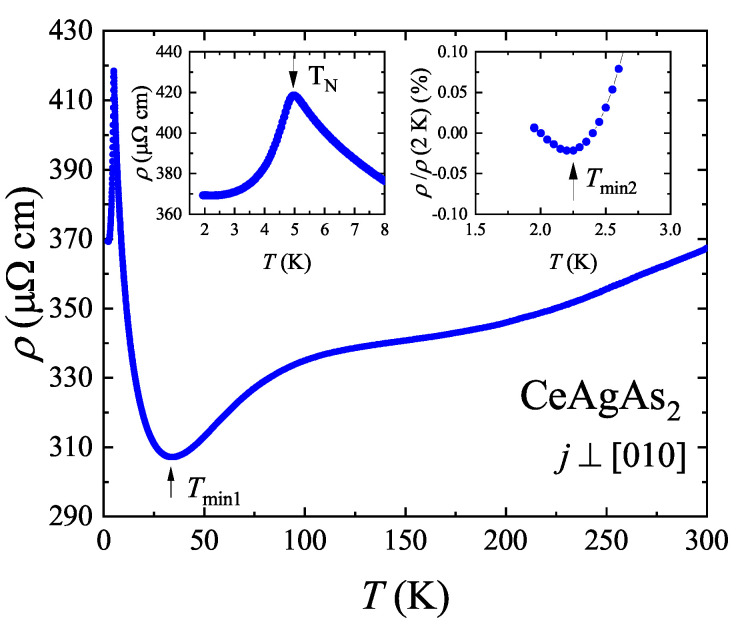
Electrical resistivity ρ(T) of single-crystalline CeAgAs_2_ measured within the crystallographic (010) plane. The insets present the low-temperature data highlighting the maximum of the resistivity at $T_{N}$ (left-hand side) and the minimum at $T_{\min2}$ (right-hand side).

The temperature variation of the electrical resistivity ρ(T) of CeAgAs_2_ was measured with electric current flowing within the crystallographic (010) plane. The $\rho_{⊥}\left(T\right)$ dependence presented in Figure 6 can be divided into a few ranges. Between the room temperature and the resistivity minimum located near $T_{\min1}$ = 34 K, the resistivity decreases with decreasing temperature initially in a quasi-linear manner and then forms a broad hump around 200 K. While the former temperature dependence likely originates principally from scattering of conduction electrons on phonons, the broad anomaly in ρ(T) may be attributed to an interplay between CEF interactions and Kondo effect usually observed for Ce-based intermetallics [20]. Below $T_{\min1}$ the resistivity sharply increases in a manner typical of insulators or semiconductors. Remarkably, this semiconducting-like change of the resistivity is interrupted at $T_{\max}$ = 5 K, where ρ reaches its maximum value (see the left-hand side inset to Figure 6). The peak position matches very well with the AFM transition temperature $T_{N}$ derived from the magnetic susceptibility measurements. Below $T_{N},$ the resistivity decreases again, which is mainly a result of scattering of charge carriers on magnons. Interestingly, the expected tendency towards saturation of the resistivity at temperatures much lower than $T_{N}$ is broken near $T_{\min2}$ = 2.3 K, below which a small upturn occurs (see the right-hand side inset to Figure 6). However, as argued below, the origin of this increase is different from that of the resistivity behavior between $T_{\min1}$ and $T_{N}$.

**Figure 7:**
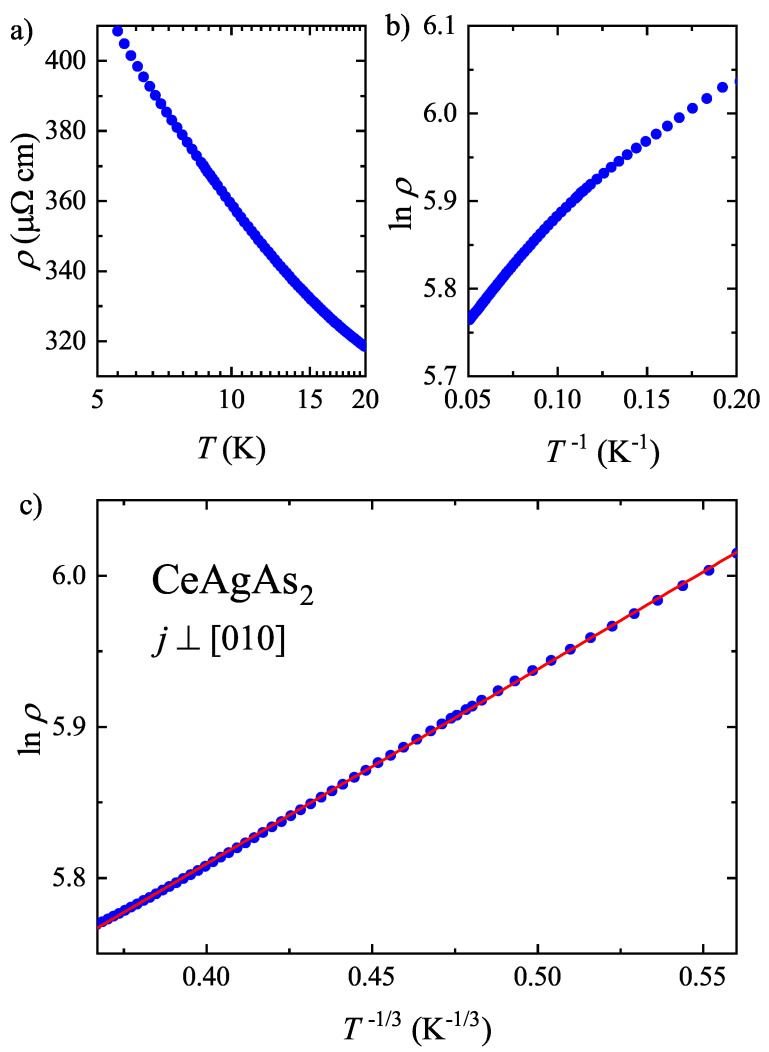
Different scaling relations of the resistivity data of CeAgAs_2_, measured in the range 5.5–20 K as described in the caption of Figure 6, appropriate for (**a**) incoherent Kondo scattering, (**b**) semiconducting activation of charge carriers across energy gap, (**c**) variable range hopping of electrons between localized in-gap states. Solid line represents the function $\rho \left(T\right)=\rho_{H}\exp\left[{\left(T_{H}/T\right)}^{x}\right]$ with x=1/3.

In order to find out what kind of scattering mechanism is responsible for the electrical transport in CeAgAs_2_ before the AFM order sets in, the ρ(T) data measured in the range of 5.5–20 K were analyzed in terms of three different physical scenarios. In Figure 7a, a logarithmic temperature dependence of the resistivity is examined, characteristic of incoherent Kondo scattering that was suggested in Ref. [12] to dominate the charge transport in CeAgAs_2_ above $T_{N}$. Apparently, in the case of the crystal studied herein, a ρ∝lnT behavior can hardly be identified, which indicates that the simple Kondo scenario may not be adequate. In this context, it should be noted that the magnitude of the resistivity of CeAgAs_2_ increases within narrow temperature interval (between 34 and 5 K) by more than 100 μΩ cm. Such a semiconducting-like character may be ascribed to the formation of a small pseudo-gap at the Fermi energy in CeAgAs_2_, as revealed in band structure calculations (see below). Typically, the resistivity can be approximated by the expression $\rho \left(T\right)=\rho_{0}\exp\left(\Delta /T\right)$ describing thermal excitation of conduction electrons over hybridization gap Δ.

As can be inferred from Figure 7b, the $\ln\rho (T^{-1})$ dependence does not reproduce well the resistivity data of CeAgAs_2_. Anticipating the presence of several active channels of the electrical conduction, such as transport involving in-gap donor or acceptor states, the resistivity of CeAgAs_2_ in the same temperature range from 5.5 K to 20 K was analyzed in terms of the relation $\rho \left(T\right)=\rho_{H}\exp\left[{\left(T_{H}/T\right)}^{x}\right]$, where *x* is a parameter which defines different hopping transport regimes and $\rho_{H}$ represents a characteristic material constant [21,22]. This type of temperature dependence of the resistivity is basically expected in insulators and semiconductors, in which conduction of electric current is realized through hopping of charge carriers between localized states. As shown in Figure 7c, the best description of the experimental data was obtained with the parameter *x* equal to 1/3. This finding agrees very well with the results of band structure calculations, which revealed a predominant 2D character of the Fermi surface in CeAgAs_2_ (see below).

**Figure 8:**
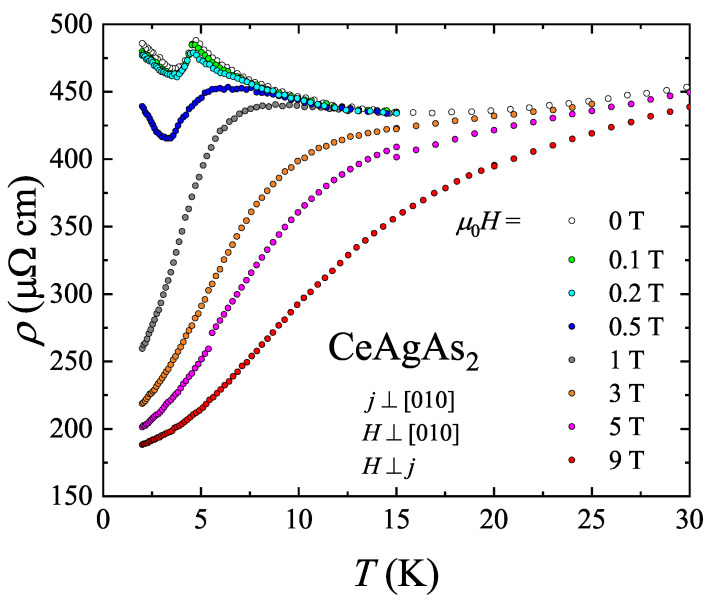
Low-temperature dependencies of the electrical resistivity of single-crystalline CeAgAs_2_ measured with electric current flowing in the orthorhombic (010) plane and magnetic field of different strength applied perpendicular to it yet confined in the same plane.

**Figure 9:**
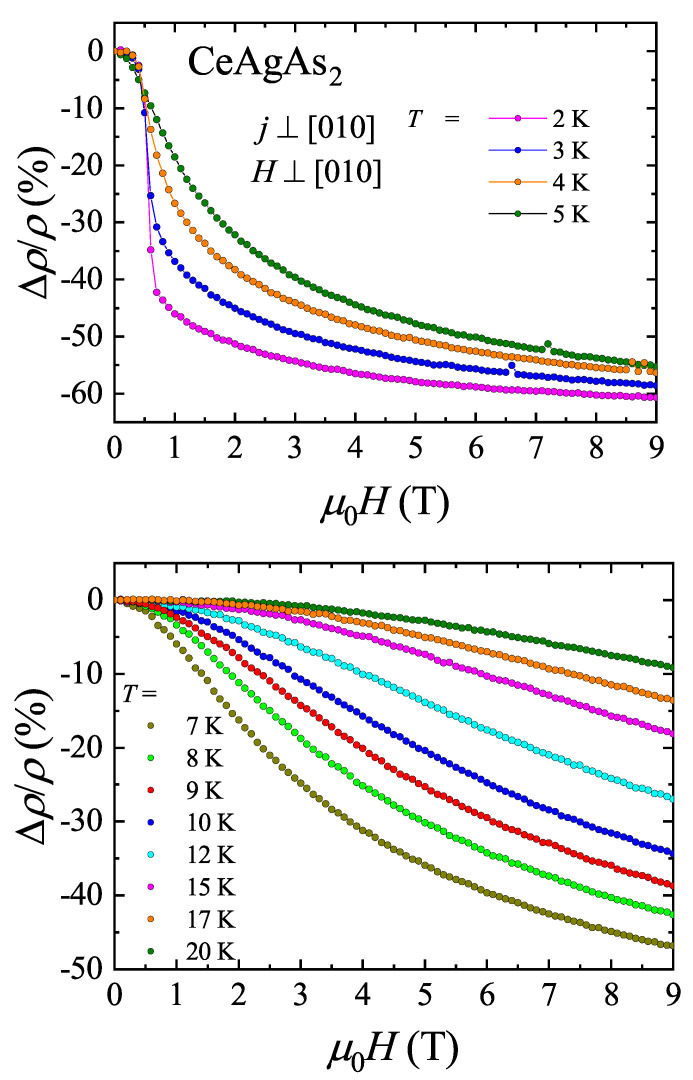
Transverse magnetoresistance isotherms of single crystalline CeAgAs_2_ measured with j⊥[010] and H⊥[010] in the AFM state (**upper panel**) and the paramagnetic state (**lower panel**).

Figure 8 displays the electrical resistivity of CeAgAs_2_ measured within the (010) plane in external magnetic field also confined in the same plane and oriented perpendicular to the flowing current. These measurements were carried out using different single-crystal of CeAgAs_2_ from the same batch. It should be noted that the resistivity of this crystal is somewhat larger, the low-temperature minimum in ρ(T) is located at higher temperature and the lowest-temperature upturn is more significant compared to the data presented in Figure 6. As can be seen in the figure, in weak magnetic fields, the electrical resistivity is hardly changed, and the main effect is small shift of the maximum due to magnetic ordering towards lower temperatures, as expected for antiferromagnets. However, in a field 0.5 T, the decrease of the resistivity is distinct and the maximum is much broader. At stronger fields, the latter feature disappears completely and the resistivity decreases significantly. The observed behavior of ρ(T) results from the MM transition that occurs in $H_{c}=0.5$ T. Further support for this interpretation comes from the inspection of the isotherms of the transverse magnetoresistance (MR) of CeAgAs_2_ defined as $\Delta \rho /\rho =\frac{\rho (H)-\rho (0)}{\rho (0)}$ and measured with electrical current and magnetic field oriented as described above. The variations measured at low temperatures are gathered in the upper panel of Figure 9. Noticeably, initially the magnetic field hardly affects the resistivity, however in a field of 0.5 T, a sudden drop of Δρ/ρ occurs, marking the MM transition. At 2 K, the MR attains a large value of −60 % in a field of 9 T.

**Figure 10:**
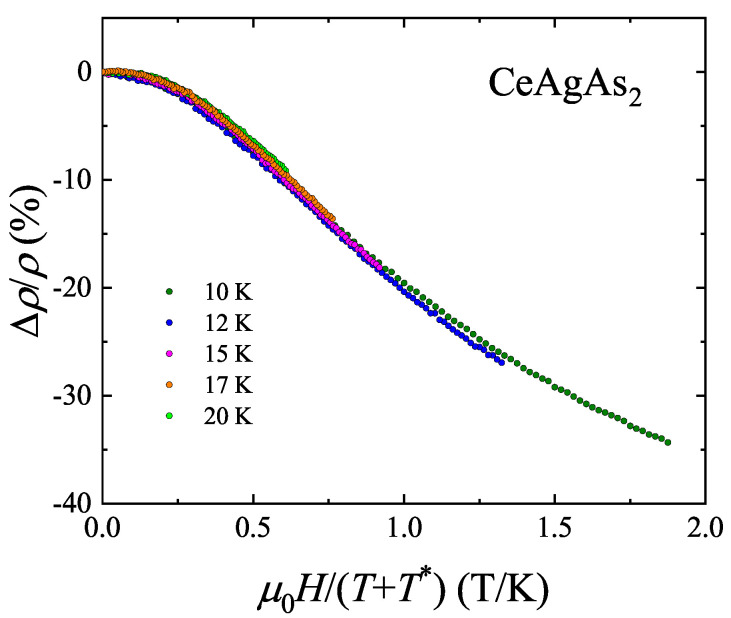
Single-ion Kondo scaling of the transverse magnetoresistance of single crystalline CeAgAs_2_ measured with j⊥[010] and H⊥[010].

The lower panel of Figure 9 presents the Δρ/ρ(H) data collected in the paramagnetic state. At each temperature the MR is negative and large. As displayed in Figure 10, all the isotherms can be superimposed onto each other using the single-ion Kondo scaling relation $\Delta \rho /\rho \left(H\right)=f\left(\frac{H}{T+T^{★}}\right)$, derived within the Bethe–Ansatz approach [23]. The characteristic temperature $T^{★}$, which ensures the best overlap of the MR curves, amounts to −5.2 K. This value is close to that given in Ref. [12]. The negative sign of $T^{★}$ manifests strong ferromagnetic interactions between magnetic moments within the (010) plane, as revealed by neutron diffraction measurements [11].

**Figure 11:**
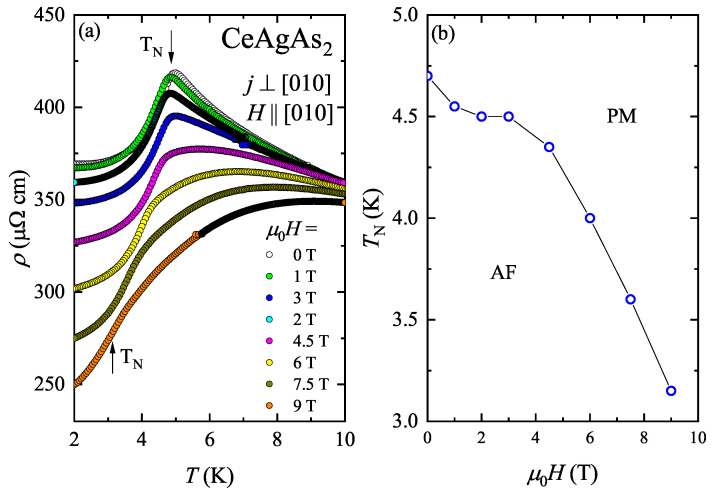
(**a**) Temperature dependence of the resistivity of CeAgAs_2_ measured in the vicinity of $T_{N}$ in different magnetic fields. (**b**) Néel temperature as a function of magnetic field.

To elucidate the source of the low-temperature upturn in the electrical resistivity, the resistivity measurements were performed with magnetic field oriented along the [010] axis, being hard magnetic direction. Influence of the magnetic field on the ρ(T) dependencies of CeAgAs_2_ measured in the vicinity of $T_{N}$ is shown in Figure 11a. In magnetic fields up to 3 T, ρ(T) variation does not change significantly. With increasing the field strength, the peak in ρ(T) shifts slightly towards lower temperatures while the magnitude of the resistivity diminishes giving rise to negative MR. Above 3 T, this sharp peak transforms into a broad maximum which moves towards higher temperatures with increasing field. The evolution of the Néel temperature, defined as a maximum in the first derivative of the resistivity dρ/dT with magnetic field is shown in Figure 11b.

**Figure 12:**
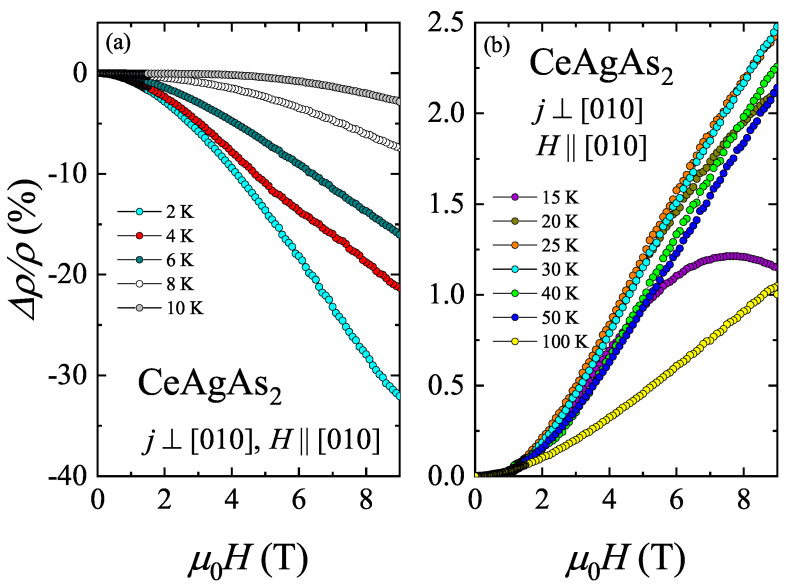
(**a**) The transverse magnetoresistance of CeAgAs_2_ measured as a function of magnetic field in the temperature intervals 2–10 K, and (**b**) 15–100 K (right-hand-side panel).

Figure 12 depicts the magnetic field dependence of the MR taken with external magnetic field oriented along the [010] axis, i.e., perpendicular to the magnetic moments which are confined within the crystallographic (010) plane [11]. Between 2 and 10 K, Δρ/ρ is negative in the entire field range. The absolute value of Δρ/ρ is quite significant at the lowest temperatures being equal to −32 % at 2 K and in a field of 9 T. Remarkably, at this temperature, no MM-like anomaly in Δρ/ρ(H) was found up to 9 T. With increasing temperature, the absolute value of the MR decreases. At T = 4 K, a small hump is seen near 6 T that can be attributed to the MM transition. This behavior is in line with the data presented in Figure 11b. For T⩾ 15 K, the MR changes sign to positive. At 15 K, the negative contribution to Δρ/ρ can be still observed at higher fields as a decrease of the MR above 7 T. With increasing temperature, the negative contribution to Δρ/ρ decreases. It is also worth noting that the magnitude of positive MR is much smaller than the negative contribution observed at low temperatures. The maximum positive value of the order of 2.5 % was found at 25 and 30 K, i.e., in the vicinity of $T_{\min1}$. Above $T_{\min1}$, the magnitude of Δρ/ρ decreases. The positive MR observed at high temperatures usually result from cyclotron motion of conduction electrons in the presence of external magnetic field.

**Figure 13:**
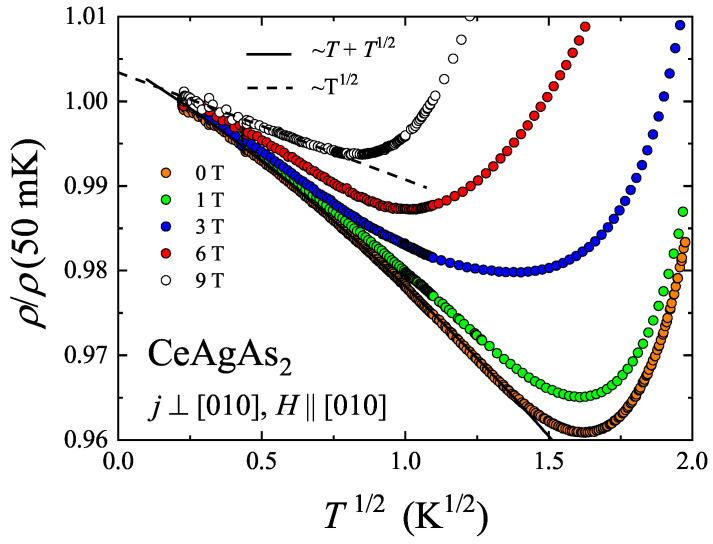
Temperature dependence of the relative resistivity of CeAgAs_2_ measured in different magnetic fields in the temperature interval 0.05–4 K. The solid and dashed lines represent $\rho =\rho_{0}-A_{C}T^{1/2}-A_{\mathrm{SW}}T$ and $\rho =\rho_{0}^{9T}-A_{C}^{9T}T^{1/2}$ fitting functions, respectively.

In order to further search for the origin of the low temperature upturn in the resistivity, the measurements were extended towards lower temperatures. Though performed on a different sample of CeAgAs_2_, the orientations of current and magnetic field with respect to the crystallographic axes were preserved, i.e., j⊥[010] and H‖[010]. Figure 13 shows the temperature variation of the resistivity normalized to its value at 50 mK (note a $T^{1/2}$ scale). The resistivity goes through a minimum near $T_{\min2}=$ 2.6 K, which is slightly higher than $T_{\min1}$ found for the other sample (see above), however the magnitude and the field dependence of Δρ/ρ of the two specimens are very similar. Between 0.1 and 2 K, no hint at any MM transition was found in accordance with the phase diagram presented in Figure 11b. It should be noted that for both single-crystalline samples of CeAgAs_2_, the resistivity attains rather high magnitude, which probably implies that elastic scattering of conduction electrons on crystal structure imperfections plays an important role in the low temperature electronic transport. Remarkably, as can be inferred form Figure 13, the upturn in ρ(T) of the sample measured in zero field can be described by the relation: $\rho =\rho_{0}-A_{C}T^{1/2}$ - $A_{\mathrm{SW}}T$ with the residual resistivity $\rho_{0}$ = 389.4 μΩ cm, and the prefactors $A_{C}$ = 8.03 μΩ cm K${}^{-1/2}$ and $A_{\mathrm{SW}}$ = 2.27 μΩ cm K${}^{-1}$. The so-derived residual resistivity is somewhat larger than that found for the sample considered above. The square-root-in-*T* term represents interaction quantum correction or Altshuler-Aronov correction, which is a typical contribution to low temperature resistivity of disordered conductors independent on their ground state properties [24,25,26]. In turn, linear-in-*T* term stands for quantum correction due to scattering of conduction electrons on spin waves [27], expected for magnetically ordered materials, being independent of the type of magnetic ordering and the system dimensionality.

As shown in Figure 13, the observed linear-in-*T* behavior gradually disappears with increasing the strength of external magnetic field. The resistivity measured in a field of 9 T can be well described below the minimum by relation containing only the square-root-in-*T* contribution: $\rho =\rho_{0}^{9T}-A_{C}^{9T}T^{1/2}$, where $\rho_{0}^{9T}$ = 241.9 μΩ cm and $A_{C}^{9T}$ = 3.05 μΩ cm K${}^{-1/2}$. This finding is in an agreement with the behavior anticipated for magnetically ordered metallic systems, in which spin-wave mediated interaction correction to the resistivity becomes suppressed in strong magnetic fields due to damping of spin waves excitations. In turn, the Coulomb interaction correction governed mainly by singlet term and triplet term with $S_{z}=0$ inherently does not depend on the magnetic field, while triplet term with $S_{z}=\pm$ 1 is cut off for fields large enough to make the Zeeman splitting energy much larger than thermal energy of conduction electrons. In such conditions the Coulomb interaction is not destroyed by magnetic field and the system exhibits positive MR.

Since the singlet and triplet terms contribute to the total quantum correction with opposite signs, one may expect that magnitude of the interaction correction does not decrease with increasing magnetic field. This same, the minimum should shift to higher temperatures. As shown in Figure 11, in a magnetic field of 9 T the residual resistivity of CeAgAs_2_ is reduced down to about 120 μΩ cm. Since the quantum corrections are correlated with the residual resistivity value, one can deduce that with decreasing the residual resistivity the coefficient $A_{C}$ decreases when magnetic field is applied. The difference between values of $A_{C}$ and $A_{C}^{9T}$ may reflect the presence of another contribution to the resistivity below the minimum with very similar temperature dependence as that of the interaction correction. A likely candidate is weak localization correction that appears in magnetically ordered materials when inelastic scattering rate due to electron–magnon scattering is much larger than that associated with electron–phonon interaction [27]. In such case, contrary to non-magnetic materials, the dephasing rate $1/\tau_{\varphi }$ is a relevant cut-off for the quantum interference phenomena. In the literature, there were considered two limiting scenarios, i.e.,: $ћ/\tau_{\varphi }\ll \Delta$ and $ћ/\tau_{\varphi }\gg \Delta$ ($\tau_{\varphi }$ and Δ are dephasing time and energy gap in magnon spectrum, respectively). In the former case the correction to the conductivity is proportional to ${\left(\pi \sinh\left(\Delta /T\right)\right)}^{1/2}$ for T>Δ or is given by fractional power law in the crossover regime T∼Δ. In turn, the correction predicted for $ћ/\tau_{\varphi }\gg \Delta$ is proportional to ${\left(T\ln\left(const/T\right)\right)}^{1/2}$. Remarkably, the two latter relations yield a similar square-root-in-*T* dependence of low temperature resistivity, experimentally observed for CeAgAs_2_.

### 2.5. Band Structure Calculations

**Figure 14:**
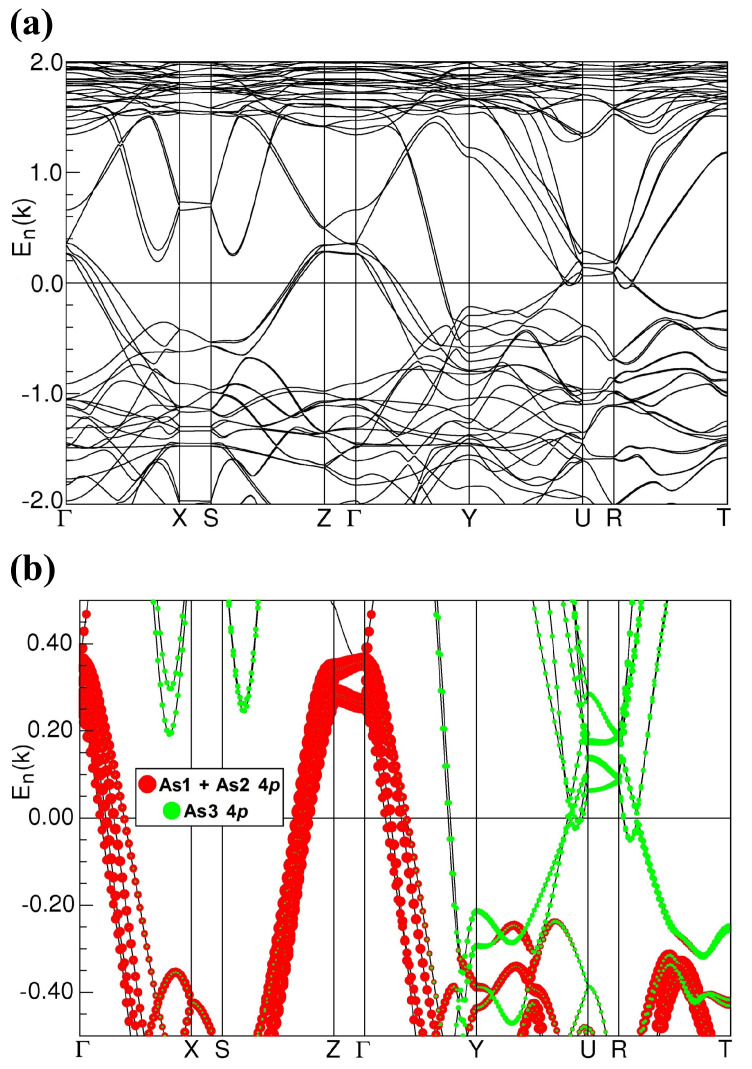
(**a**) The LSDA+U (local spin density approximation) (U = 6 eV) electronic band structure of the AFM (antiferromagnetic) ground state of CeAgAs_2_ along high-symmetry lines in the orthorhombic BZ (Brillouin zone). (**b**) The corresponding As 4*p* orbital contributions to the electronic bands (band weights) originating from different As sites in the vicinity of $E_{F}$ (zero energy).

The electronic band structure calculated for antiferromagnetic CeAgAs_2_ (see Figure 14), exhibits a metallic character and generally it is very similar to the band structure of the nonmagnetic reference compound LaAgAs_2_ (Ref. [10]). It does not show any Dirac-like bands, found for a few other rare-earth (RE) pnictides RETX_2_ with X = Sb or Bi [28,29,30,31]. Probably it is a combination of the AFM ordering (which splits the electronic bands) with the distortion of the nets of As3 atoms from perfect squares that causes the lack of conic-like bands originating from the As 4*p* electrons near the Fermi level $E_{F}$. One may consider that the quasi-two-dimensional (quasi-2D) band structure (along the [010] axis) reflected in a group of split and flat bands lying 0.05–0.2 eV above $E_{F}$ along the ΓZ and UR lines in the Brillouin Zone (BZ) mainly governs the transport properties of CeAgAs_2_.

**Figure 15:**
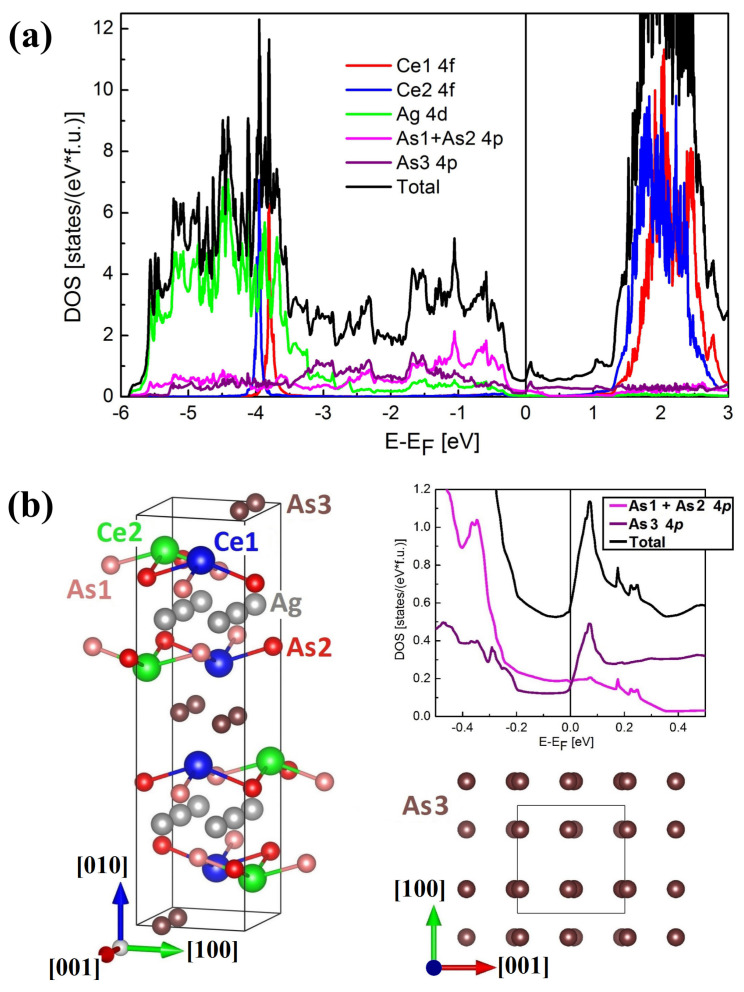
(**a**) Total and partial electronic densities of states calculated for CeAgAs_2_ in the LSDA+U (U = 6 eV) approach with the AFM ordering along the [100] axis. (**b**) Left panel: unit cell of CeAgAs_2_; right panel: enlarged DOS (density of states) in the vicinity of $E_{F}$ and the sketch of quasi-2D nets of the As atoms (As3 sites) with visible distortion from the ideal square-like shape. The structural figures were obtained using the VESTA program [38].

The total density of states (DOS) (see Figure 15) has rather a low value at $E_{F}$ ($N(E_{F})\approx 0.6$ states eV${}^{-1}$ f.u.${}^{-1}$, where f.u. means the formula unit), which leads to the estimated value of the Sommerfeld coefficient $\gamma_{\mathrm{calc}}\approx 1.4$ mJ K${}^{-2}$ mol${}^{-1}$. This value is close to γ=1.2 mJ K${}^{-2}$ mol${}^{-1}$, determined experimentally for LaAgAs_2_ [12], while an order of magnitude smaller than the experimental value 50 mJ K${}^{-2}$ mol${}^{-1}$ determined for CeAgAs_2_ (see above). The latter value of γ indicates strong electron–electron correlations typical of moderate heavy-fermion behavior. The difficulty in reproducing in band calculations proper values of γ is well-known shortcoming of DFT methods applied to such systems (see, e.g., Ref. [32]). The additional Coulomb potential U is responsible for displacement of the positions of the Ce 4*f* states. The narrow peaks due to the 4*f* electrons, which are fairly well localized near 4 eV below $E_{F}$ are a source of the AFM order in CeAgAs_2_. It is worth noting that the two different sites of Ce atoms (labeled in Figure 15b as Ce1 and Ce2) show some small differences in the position of the 4*f* DOS peaks being about 150 meV. However, the intensities of the two peaks are almost the same. Similar location of the 4f states below $E_{F}$ was observed for other magnetic Ce-based compounds [33,34]. Despite the metallic character of the electronic structure of CeAgAs_2_, the reduction of DOS around $E_{F}$ is significant, alike in other RETAs${}_{2}$ phases [10,35,36]. That feature can be a reason for the semiconducting-like behavior of CeAgAs_2_ as well as few similar arsenides with the cis-trans distortions in As3 layers [10,35]. Principally, DOS at $E_{F}$ consists of the As 4*p* contributions like in other RETX_2_ compounds [28,30,37]. However, the 4*p* contributions originating from As1 and As2 are comparable to each other and their sum is very similar to that of the As3 atomic sites at $E_{F}$. Meanwhile, in other RETX${}_{2}$ compounds the contributions of the quasi-2D square nets of pnictogen atoms (without distortions) are dominating in total DOS near $E_{F}$. The Ag 4*d* electrons contribute to total DOS in the range from −6 eV to about −3 eV below $E_{F}$, wherein both Ag atoms yield very similar shape of DOS, hence it is presented here as a sum.

In the band calculation performed for CeAgAs_2_, an antiferromagnetic arrangement (+ - - +) of ferromagnetically ordered Ce atom layers was assumed, as determined from the neutron diffraction data [11]. The suitable magnetic supercell contained four nonequivalent Ce positions (twice more than those in the non-magnetic unit cell) with sufficiently large initial values of magnetic moments. By minimizing the total energy of such a magnetic supercell in the self-consistent field calculation the following values of the magnetic moments were obtained: +1.0142, +1.0135 and −1.0142, −1.0135 $\mu_{B}$ per nonequivalent Ce site in two adjacent planes, respectively. The Ce moment of about $1.01\;\mu_{B}$ is in accord with the results of the magnetization measurements (see above) and the neutron diffraction experiment [11].

**Figure 16:**
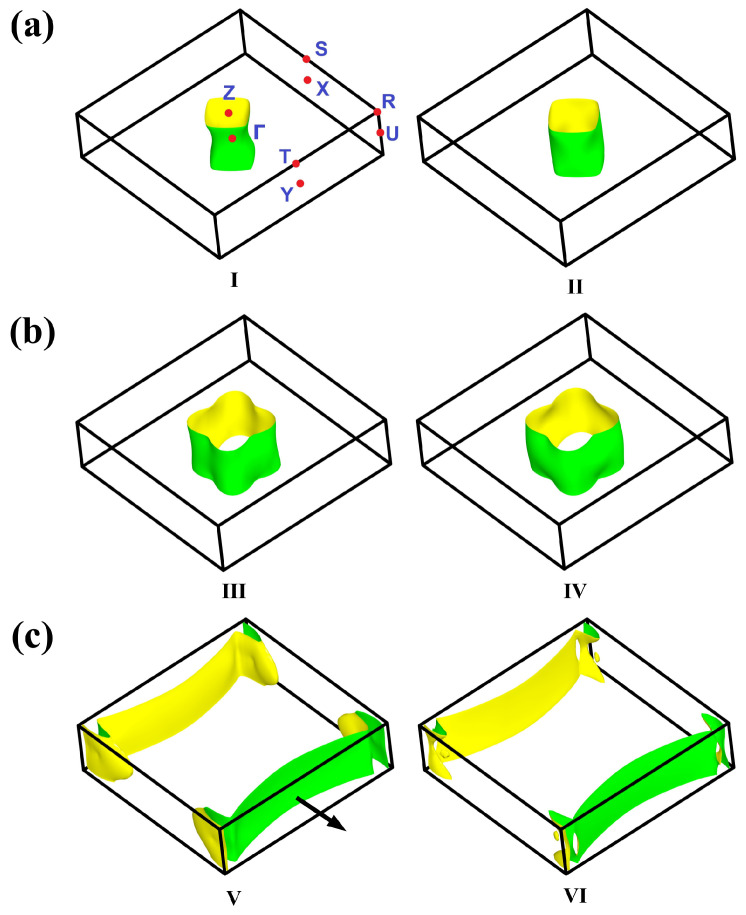
Fermi surface sheets in the AFM (antiferromagnetic) state of CeAgAs_2_ calculated within the LSDA+U (local spin density approximation) approach (U = 6 eV). The sheets of similar shapes (originated from the pairs of succeeding conduction bands numbered by increasing Roman numerals with increasing their energy) are displayed in panels (**a**–**c**). High-symmetry points in the first BZ (Brillouin zone) are highlighted in panel (**a**). The black thick arrow in panel (**c**) indicates the direction of the quantization axis [100] for the AFM ordering.

As clearly apparent from Figure 16, the three FS sheets in CeAgAs_2_, possess a quasi-2D character in concert with the strong anisotropy of the physical properties of the compound. The 4-fold symmetry of the cylindrical sheets, characteristic of the tetragonal RETX_2_ compounds [39,40] is approximately preserved (see Figure 16a,b), despite the small orthorhombic distortion present in CeAgAs_2_, and this finding supports the afore-made simplifications in the CEF analysis of the magnetic data of the compound (see above). Remarkably the two FS sheets displayed in Figure 16c exhibit apparent breaking of the 4-fold rotational symmetry into a two-fold one (nematic-like transition). The FS sheets labeled V and VI present discontinuity in the $k_{z}$ (ΓY) direction in BZ possessing quasi-1D character, which can be explained by an existence of the trans-cis distortion of the As3 atomic nets along the [001] axis. This type of Peierls-like transition usually leads to opening energy gap in the electronic band structure along the axis of distortion. As reflected in orbital characters of the electronic bands visualized in Figure 14b those forming FS V and FS VI sheets originate entirely from the As3 4*p* states (note the green band weights along the ΓX and ΓY lines).

## 3. Discussion and Conclusions

Single crystals of CeAgAs_2_, grown using chemical vapor transport method, were found to crystallize with an orthorhombic structure, which is derivative of the tetragonal HfCuSi${}_{2}$-type structure. The obtained crystals were twinned due to small difference between the *a* and *c* lattice parameters of their pseudo-tetragonal unit cell.

The physical properties measurements corroborated that CeAgAs_2_ orders antiferromagnetically at low temperatures. The antiferromagnetic ordering manifests itself as a maximum in the magnetic susceptibility, a λ-like anomaly in the specific heat, and a distinct maximum in the electrical resistivity. At odds with the recent literature data [12], a single antiferromagnetic phase transition at $T_{N}$ = 4.9 K was found. At *T* = 1.72 K, the compound exhibits a metamagnetic transformation in a magnetic field of 0.5 T oriented perpendicular to [010] axis of the orthorhombic unit cell. This transition gives rise to distinct features in the low-temperature field dependencies of the magnetization and the magnetoresistance. Furthermore, in magnetic fields stronger than 0.5 T, the temperature variations of the heat capacity and the electrical resistivity distinctly change their character. The saturated magnetic moment is equal to 1 $\mu_{B}$, in good agreement with the neutron diffraction data [11], as well as the results of band structure calculations performed with the Coulomb parameter U= 6 eV.

The band structure calculations showed that Fermi surface sheets in CeAgAs_2_ have strongly quasi-2D character. The total density of states at the Fermi energy is rather low. The Sommerfeld coefficient calculated within the free-electron formula has a value similar to that of the non-magnetic reference compound LaAgAs_2_. Remarkably, the reduced density of states at the Fermi level brings about an activation-like behavior of the electrical resistivity at low temperatures [21,22]. In parallel, the electrical transport in CeAgAs_2_ is governed at the lowest temperatures by quantum corrections occurring due to crystal structure imperfections.

## 4. Materials and Methods

Single crystals of CeAgAs_2_ were synthesized using chemical vapor transport method with iodine as a transport agent, as described elsewhere [9]. The obtained crystals had a form of thin platelets with linear dimensions up to 2 mm with their large face perpendicular to the crystallographic direction [010]. The crystal structure was investigated at room temperature on an Oxford Diffraction four-circle diffractometer equipped with a CCD camera (MoKα radiation λ=0.7107 Å). The crystal structure was solved and refined from x-ray diffraction (XRD) data employing the SHELXL program [41] (own structure type, being a HfCuSi_2_ derivative, space group *Pbcm*, lattice parameters *a* = 5.7797(2) Å, *b* = 21.0418(6) Å, *c* = 5.7682(2) Å).

Dc magnetic measurements were performed within the temperature interval 1.72–400 K and in magnetic fields up to 5 T using a Quantum Design MPMS-5 superconducting quantum interference device (SQUID) magnetometer. The crystal was mounted on an acrylic glass support. Due to small mass of crystals, first the magnetization of the support was measured and then this contribution was subtracted from the total SQUID response. The heat capacity was measured from 0.4 to 100 K and in magnetic fields up to 9 T employing a relaxation technique. Electrical transport measurements were made in the temperature range 50 mK–300 K and in magnetic fields up to 9 T using a standard ac four-probe method. The heat capacity and electrical resistivity experiments were carried out using a Quantum Design PPMS-9 platform.

Band structure calculations were performed with the full-potential local-orbital (FPLO-14) code within density functional theory (DFT) [42]. The Perdew–Wang parameterization (PW92) [43] of the local spin density approximation (LSDA) was used in scalar relativistic mode. The experimentally determined lattice parameters and atomic positions (see Section 2.1) and the AFM ground state obtained in the neutron diffraction measurements [11] with the magnetic moments arrangement along the [100] axis were assumed. In addition, a Coulomb repulsion potential U = 6 eV in the LSDA+U approach [44] for two different Ce sites was applied. Sets of the valence basis were automatically selected by the internal procedure of FPLO-14. Total energy values were converged with accuracy to 1 meV for the 12 × 12 × 12 **k**-point mesh, corresponding to 588 **k**-points in the irreducible part of the orthorhombic Brillouin zone (BZ). The Fermi surface (FS) sheets were calculated for a much denser grid of 27720 **k**-points.

## Figures and Tables

**Table 1:** Crystallographic and structure refinement data for CeAgAs_2_.

Compound	CeAgAs_2_
Space group	*Pbcm*
	*a* = 5.7797(2) Å
	*b* = 21.0418(6) Å
Unit cell dimensions	*c* = 5.7682(2) Å
Volume	701.50(4) Å${}^{3}$
Formula weight	397.83 g/mol
Calculated density	7.53 g/cm${}^{3}$
Absorption coefficient	36.835 mm${}^{-1}$
θ range for data collection	3.511°–37.353°
	−9⩽h⩽9
	−35⩽k⩽36
Ranges in *hkl*	−9⩽l⩽9
Reflections collected/unique	31905/1528
Completeness to θ = 36.23°	99.9%
Refinement method	Full-matrix least-squares on F2
Refined parameters	46
Goodness of fit on F${}^{2}$	1.102
Final R indices [I≥2σ(I)]	R1 = 0.0293, wR2 = 0.0593
R indices (all data)	R1 = 0.041, wR2 = 0.0643
Extinction coefficient	0.00093(4)
Largest diff. peak and hole	3.776 and −2.016 e/Å${}^{-3}$

**Table 2:** Atomic coordinates and equivalent isotropic thermal displacement parameters for CeAgAs_2_. $U_{eq}$ is defined as one third of the trace of the orthogonalized $U_{ij}$ tensor. All atomic sites are fully occupied.

Atom	Site	*x*	*y*	*z*	$U_{\mathrm{eq}}$$(10^{-3}$Å${}^{2})$
Ce1	4*d*	0.51183(8)	0.61363(2)	0.25	8(1)
Ce2	4*d*	0.01274(8)	0.88214(2)	0.25	8(1)
Ag1	4*c*	0.23826(10)	0.25	0.5	12(1)
Ag2	4*c*	0.73584(11)	0.25	0.5	12(1)
As1	4*d*	0.51271(13)	0.83859(3)	0.25	9(1)
As2	4*d*	0.01242(13)	0.66212(3)	0.25	9(1)
As3	8*e*	0.22338(10)	0.00075(4)	0.52628(10)	13(1)

**Table 3:** Anisotropic thermal displacement parameters for the atoms in CeAgAs_2_ (in 10${}^{-3}$Å${}^{2}$). The anisotropic displacemant factor exponent takes the form: $-2\pi^{2}\left[h^{2}a^{*2}U_{11}+\ldots +2hka^{*}b^{*}U_{12}\right]$.

	$U_{11}$	$U_{22}$	$U_{33}$	$U_{12}$	$U_{23}$	$U_{13}$
Ce1	7(1)	8(1)	10(1)	0.2(1)	0	0
Ce2	7(1)	8(1)	10(1)	0.3(1)	0	0
Ag1	12(1)	11(1)	14(1)	0	0.5(2)	0
Ag2	11(1)	11(1)	14(1)	0	0.9(2)	0
As1	7(1)	10(1)	10(1)	0	0	0
As2	7(1)	10(1)	10(1)	0	0	0
As3	14(1)	8(1)	17(1)	0.4(2)	2(1)	0.5(2)
